# Vitamin D Status Determines the Effect of Cabergoline on Sexual Function and Depressive Symptoms in Hyperprolactinemic Women

**DOI:** 10.3390/nu17111813

**Published:** 2025-05-27

**Authors:** Robert Krysiak, Karolina Kowalcze, Johannes Ott, Andrea Deledda, Bogusław Okopień

**Affiliations:** 1 Department of Internal Medicine and Clinical Pharmacology, Medical University of Silesia, Medyków 18, 40-752 Katowice, Poland; bokopien@sum.edu.pl; 2 Department of Pediatrics in Bytom, Faculty of Health Sciences in Katowice, Medical University of Silesia, Stefana Batorego 15, 41-902 Bytom, Poland; kkowalcze@sum.edu.pl; 3 Department of Pathophysiology, Faculty of Medicine, Academy of Silesia, Rolna 43, 40-555 Katowice, Poland; 4 Clinical Division of Gynecologic Endocrinology and Reproductive Medicine, Department of Obstetrics and Gynecology, Medical University of Vienna, 1090 Vienna, Austria; johannes.ott@meduniwien.ac.at; 5 Endocrinology and Obesity Unit, Department of Medical Sciences and Public Health, University of Cagliari, 09124 Cagliari, Italy; andredele@tiscali.it

**Keywords:** mood, prolactin excess, reproductive axis, sexual dysfunction, vitamin D homeostasis, women’s health

## Abstract

*Background/Objectives*: Although untreated prolactin excess is often associated with female sexual dysfunction, sexual functioning improves after chronic administration of dopamine agonists, including cabergoline. Extra-sexual benefits of cabergoline therapy were found to be less pronounced in young hyperprolactinemic women in the case of coexistent hypovitaminosis D. Thus, the present study was aimed at investigating whether vitamin D status also determines cabergoline action on sexual function and depressive symptoms in reproductive-age women. *Methods*: This prospective cohort study included 75 young women with prolactin excess, who, depending on vitamin D status, were assigned to one of three groups. Females with vitamin D deficiency (group A), vitamin D-insufficient women (group B) and vitamin D-sufficient women (group C) were matched for age, body mass index, blood pressure and prolactin levels. For the following six months, they received cabergoline. Before and after cabergoline treatment, all participants completed questionnaires evaluating female sexual functioning (FSFI) and depressive symptoms (BMI-II). The remaining outcomes of interest included plasma levels of 25-hydroxyvitamin D, prolactin and sex hormones. *Results*: Before treatment, there were no differences between the study groups in sexual functioning and mood. The study groups differed in post-treatment levels of 25-hydroxyvitamin D, prolactin, testosterone and estradiol. Although cabergoline reduced the total FSFI score and improved functioning in all domains of the FSFI questionnaire, this effect was strongest in group C and weakest in group A. Statistically significant changes in the BDI-II score were observed only in group C. The increase in the total FSFI score and domain scores correlated with the decrease in prolactin levels, 25-hydroxyvitamin D levels, the increase in testosterone and estradiol concentrations, and the reduction in the BDI-II score. *Conclusions:* Low vitamin D status attenuates the beneficial effects of cabergoline on sexual function and depressive symptoms in reproductive-age women.

## 1. Introduction

Hyperprolactinemia, defined as prolactin levels exceeding the upper normal limit, is considered the most common endocrine disorder of the hypothalamic-pituitary axis and has multifactorial etiology [1]. The presenting symptoms of prolactin excess in reproductive-age women include menstrual disorders (amenorrhea or oligomenorrhea), galactorrhea (spontaneous or provoked), delayed menarche and infertility [2]. Unlike men, in whom hypolibidemia and erectile dysfunction are typical features of hyperprolactinemia, the sexual functioning of women with elevated prolactin levels has attracted much less interest. Lundberg and Hulter [3] were the first who reported that impaired sexual desire was diagnosed more frequently in women with a documented organic hypothalamo–pituitary disorder in case of elevated (62.4%) than normal prolactin levels (32.6%). In a subsequent study, Kadioglu et al. [4] observed that prolactin excess impaired all aspects of female sexual functioning: desire, arousal, lubrication, orgasm, satisfaction and pain. Very similar results were obtained by Krysiak et al. [5], who additionally observed that the degree of sexual dysfunction was impacted by the molecular size of prolactin. Unlike severe sexual dysfunction induced by an excess of the monomeric hormone, an isolated increase in high-molecular-weight isoforms of prolactin (macroprolactinemia) was associated only with reduced libido [5]. Lastly, in women with polycystic ovary syndrome, prolactin concentration, though not elevated, inversely correlated with the frequency and quality of orgasm [6]. The beneficial effect on sexual functioning in women with prolactin excess was caused by dopamine agonists, which are the drugs of choice for prolactin-secreting tumors and symptomatic hyperprolactinemia of other origin [7]. Bromocriptine treatment improved all domains of FSFI, and this effect correlated with the impact on prolactin levels [8]. However, post-treatment orgasmic function and sexual satisfaction were still slightly disturbed [8]. The second dopamine agonist, cabergoline, was superior to bromocriptine. Replacement of bromocriptine with cabergoline in women poorly tolerating the former drug improved sexual drive and arousal, which might have been associated with a further decrease in plasma prolactin [9]. It seems, however, that the relationship between sexual functioning and prolactin concentration in cabergoline-treated women may be inversely U-shaped because hypoprolactinemia caused by chronic administration of inadequate high doses of this drug was complicated by impaired sexual drive and impaired sexual arousal, not observed in cabergoline-treated women with prolactin levels within the reference range [10].

More and more evidence suggests that women’s sexual health may also be affected by disturbances in vitamin D homeostasis. Reproductive-age women with low vitamin D status were characterized by sexual dysfunction, the degree of which inversely correlated with 25-hydroxyvitamin (25OHD) levels [11,12]. In both studies, sexual functioning was more disturbed in vitamin D-deficient than vitamin D-insufficient women [11,12]. Circulating levels of 25OHD in young women complaining of sexual dysfunction were lower than in their counterparts with normal sexual activity [13]. However, 25OHD did not correlate with FSFI domain scores in women with sexual dysfunction and polycystic ovary syndrome [14]. In most studies, women benefited from the administration of exogenous vitamin D. Vitamin D supplementation in young women with sexual dysfunction improved sexual functioning, and this beneficial effect was accompanied by an increase in both 25OHD and testosterone levels [15]. Exogenous vitamin D was superior to selenomethionine and myo-inositol in reducing the total FSFI score and in improving sexual desire and arousal in euthyroid reproductive-age women with autoimmune thyroiditis [16]. However, women with sexual dysfunction and polycystic ovary syndrome did not benefit if they received 15 µg (600 IU) of exogenous vitamin D [14]. Combination therapy of women aged between 40 and 60 years with vitamin D, calcium, isoflavones and inulin had a beneficial effect on desire, orgasm, pain, and sexual aspects of quality of life [17]. Vaginal suppositories containing vitamin D improved sexual functioning after menopause [18]. In postmenopausal women, topical, but not oral, vitamin D led to a significant improvement of the arousal subscale of FSFI [19]. In all mentioned studies, participants received either vitamin D_3_ preparations [15,16,18] or the form of this vitamin was not clearly specified [17,19]. No study assessed the effect of exogenous vitamin D_2_.

Considering the association between hyperprolactinemia and low vitamin D status and sexual response in women, concomitant presence of both disorders may theoretically impair female sexual functioning to a greater degree than each of these conditions alone. As far as we know, only two previous studies investigated the association between vitamin D homeostasis and treatment with dopamine agonists. Bromocriptine reduced elevated levels of calcitriol in individuals with acromegaly [20]. In turn, the impact of cabergoline on glucose homeostasis, plasma lipids and non-lipid risk factors was more pronounced in young women with mild or moderate hyperprolactinemia and normal vitamin D homeostasis than in age- and prolactin concentration-matched women with vitamin D deficiency [21]. The lack of dedicated studies encouraged our team to investigate whether vitamin D status determines sexual function and mood in young women receiving cabergoline treatment.

## 2. Materials and Methods

The study protocol was designed according to the principles of the 1975 Declaration of Helsinki and its later revisions. It was checked and approved by the institutional committee on human research. All patients gave their written informed consent after learning of the risks, benefits and other important information relevant to the study. Due to its nature, the study protocol did not require registration in a public trial registry.

### 2.1. Study Population

The study population was recruited among young women (aged 18 to 45 years) with new-onset and untreated mild-to-moderate hyperprolactinemia, defined as prolactin levels on two different occasions in the range between 30 and 100 ng/mL. Potential participants were not considered for enrollment if they had prolactin-secreting macroadenomas, mixed pituitary tumors, macroprolactinemia, any other chronic diseases, psychiatric problems, premature or early menopause, developmental or acquired anomalies of the reproductive system, sexual inactivity, homosexual or bisexual orientation, undergone urogynecological operations that might have affected sexual function, were pregnant or breastfeeding, or received any treatment (with the exception of vitamin D supplements).

Based on 25OHD levels, eligible patients were enrolled into one of three study cohorts: vitamin D-deficient women (group A), vitamin D-insufficient women (group B) and vitamin D-sufficient women (group C). Vitamin D deficiency, vitamin D insufficiency and normal vitamin D status were defined as 25OHD levels between 10 and 20 ng/mL (between 25 and 50 nmol/L), between 20 and 30 ng/mL (between 50 and 75 nmol/L), and between 30 and 60 ng/mL (between 75 and 150 nmol/L), respectively [22]. For ethical reasons, individuals with 25OHD below 10 ng/mL (below 25 nmol/L) were not considered for enrollment, and group A included only females refusing additional vitamin D therapy.

**Figure 1:**
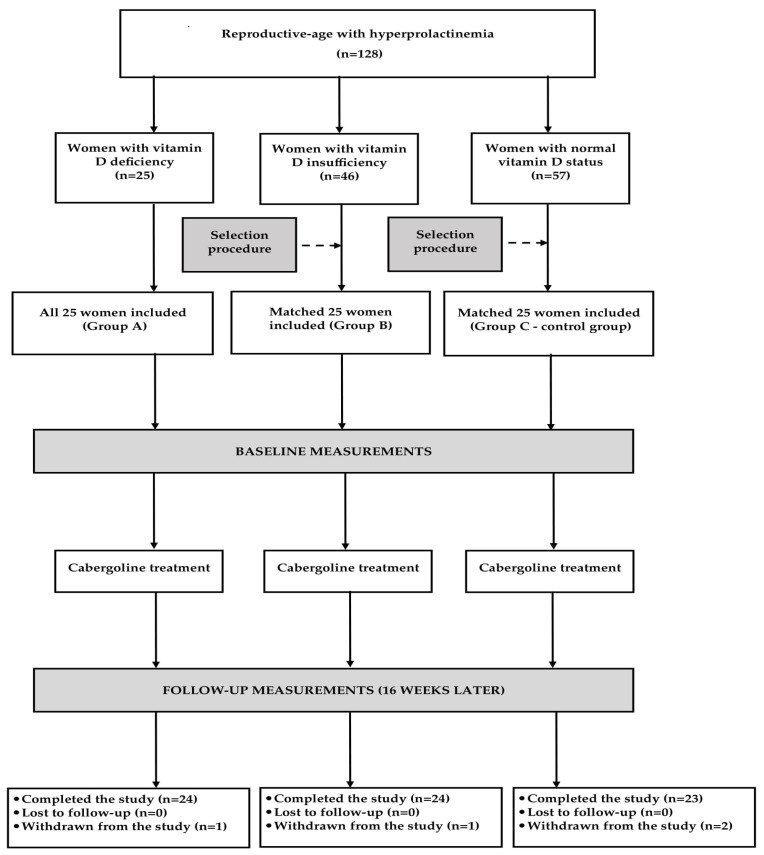
Flow the patients through the study.

Except for group A, the study groups were chosen from a larger number of eligible candidates using a computer algorithm (Figure 1). This selection was aimed at including three populations matched for age, body mass index, blood pressure and prolactin levels. A calculation performed before conducting the study showed that at least 22 patients per group must have been included to detect a 20% inter-group difference in the total FSFI score (the primary endpoint) with an 80% power, given a type I error probability of 0.05. To compensate for possible withdrawals and losses to follow up, the sample size was increased to 25 women per group. To minimize the possible influence of seasonal fluctuations in the outcome variables [23,24,25,26,27], similar proportions of women were recruited in each season (25% between January and March, 24% between April and June, 24% between July and September, and 27% between October and December).

### 2.2. Study Design

For the following six months, all women were treated with cabergoline. The drug was up-titrated from a starting dose of 0.25 mg once weekly in the first two weeks, to 0.25 mg twice weekly from the third week onwards. Cabergoline was administered at bedtime with a glass of water. The daily vitamin D intake was calculated based on analysis of individual dietary questionnaires completed by the participants throughout the study. In women taking vitamin D_3_ supplements, the total intake was obtained by summing intake from food and supplements. To exclude the possibility of interactions with this drug, vitamin D_3_-containing supplements were taken in the morning. All patients also received standard dietary advice focused on following a healthy eating plan. Adherence to the medication regimen was measured by pill count and patient report. The accepted co-treatment was transient treatment (for less than 10 days and not in the last study month) with non-steroidal anti-inflammatory drugs, acetaminophen, antimicrobials, cough suppressants, laxatives, antidiarrheal drugs or hypnotics. The withdrawal criteria included other changes in the medication regimen, consent withdrawal, cabergoline-induced hypoprolactinemia, serious adverse effects, sudden hospitalization and poor medication adherence.

### 2.3. Laboratory Assays

All measurements were performed before cabergoline treatment and again six months later. Venous blood was obtained from the antecubital vein in the follicular phase in standard conditions (collection between 8.00 and 9.30 a.m., 12 h overnight fasting, air-conditioned room) after the patient had been resting in a seated position for a minimum of 30 min. Plasma concentrations of total 25OHD, prolactin, testosterone, dehydroepiandrosterone sulfate (DHEAS) and estradiol were assayed by direct chemiluminescence using acridinium ester technology (ADVIA Centaur XP Immunoassay System, Siemens Healthcare Diagnostics, Munich, Germany). Because prolactin secretion is pulsatile [2] and increases in response to stress and venipuncture [28], its concentrations were assessed in three blood samples taken at 20 min intervals, and the obtained results were averaged. All measurements were carried out in duplicate to confirm the reproducibility of the results by persons unaware of the individual’s affiliation to the study group. The intra-assay coefficients of variation were 4.8% for 25OHD, 3.5% for prolactin, 4.6% for testosterone, 5.3% for DHEAS and 4.2% for estradiol. The inter-assay coefficients of variations were 7.6% for 25OHD, 5.2% for prolactin, 5.9% for testosterone, 7.1% for DHEAS and 6.8% for estradiol. The limits of detection were 3.2 ng/mL for 25OHD, 0.65 ng/mL for prolactin, 0.25 nmol/L for testosterone, 0.1 μmol/L for DHEAS and 28 pmol/L for estradiol. The reference ranges for the follicular phase of the cycle in our laboratory were 30–60 ng/mL for 25OHD, 5–30 ng/mL for prolactin, 0.65–3.14 nmol/L for testosterone, 2.05–10.0 µmol/L for DHEAS and 68–530 pmol/L for estradiol.

### 2.4. Questionnaires

After blood samples had been collected, all participants were invited to complete three questionnaires. This procedure took place in a separate room without the presence of any member of the research team. At the time of answering the questions, neither the participants nor the investigators were aware of the biochemical results.

The first questionnaire was aimed at gathering general information on the patient’s characteristics (age, smoking habits, physical activity, education, occupational activity, sexual partners, marriages, deliveries, miscarriages and stress exposure).

The Female Sexual Function Index (FSFI), which was the second questionnaire, is a validated, 19-item self-report scale that assesses sexual function of women over the past four weeks [29]. This questionnaire has been validated in women with hypoactive sexual desire disorder, female sexual arousal disorder, female sexual orgasm disorder, dyspareunia/vaginismus and multiple sexual dysfunctions [30]. The FSFI consists of six dimensions: desire (questions 1 and 2), arousal (questions 3–6), lubrication (questions 7–10), orgasm (questions 11–13), satisfaction (questions 14–16) and pain (questions 17–19). Each answer is rated on a scale ranging from 0 (no sexual activity) to 5 for desire and satisfaction or from 1 to 5 for the remaining domains. The score for each domain is multiplied by a domain factor (0.6 for desire, 0.3 for arousal and lubrication, and 0.4 for orgasm, satisfaction, and pain), and the result corresponds to a weighted score. The total FSFI score is obtained by summing the weighted scores for all dimensions. The total scores range from 2 to 36, with a higher score indicating better sexual function. Sexual dysfunction is defined as the overall FSFI score of less than 26.55 [29]. 

The third questionnaire, the Beck Depression Inventory Second Edition (BDI-II), is a 21-item inventory that comprises somatic, cognitive, and affective symptoms related to depression in the last two weeks [31]. These items were designed to capture the depression as defined by the American Psychiatric Association [32]. Answers are rated on a scale from 0 (not present) to 3 (severe). The responses are then summed to create a total score, with higher scores indicating greater severity of depressive symptoms [32]. The total BDI-II score can range from 0 to 63. A normal mood is characterized by a BDI-II score from 0 to 13, mild depression by a score from 14 to 19, moderate depression by a score from 20 to 28 and severe depression by a score of 29 to 63 [32].

### 2.5. Statistical Analysis

All continuous variables were log-transformed to achieve homogeneity of variance and to minimize the impact of outliers. Inter-group comparisons were performed by repeated measures analysis of variance, with post-hoc analysis carried out using Bonferroni’s test. Within-group comparisons were made using Student’s paired *t*-tests. For the categorical variables, the chi-square test was used. Bivariate relationships between the outcome variables were analyzed using Pearson’s r tests. We also carried out multivariate regression with the behavioral scores as dependent variables and the measured biochemical parameters as independent variables. The statistical significance was defined as a two-sided *p*-value below 0.05.

## 3. Results

One patient from group C was withdrawn owing to nausea, vomiting, dizziness and drowsiness that disappeared after cabergoline treatment cessation. Two women, one from group A and one from group B, prematurely terminated the study because they required chronic treatment with hypotensive and lipid-lowering drugs. Lastly, one patient from group C was withdrawn because of poor treatment adherence. There were no cases of cabergoline-induced hypoprolactinemia. Thus, the statistical analysis included 71 women (95%) who completed the study and were classified as adherent to cabergoline treatment. A post-hoc calculation showed that our analysis had sufficient power (83%).

### 3.1. General Characteristics of the Study Groups

At study entry, the groups did not differ in terms of in terms of age, body mass index, smoking habits, reasons for hyperprolactinemia, physical activity, education, occupational activity, type of work, the number of sexual partners, the number and duration of marriages, the number of deliveries and miscarriages, stress exposure, or blood pressure. There were between-group differences in total daily vitamin D intake and the percentage of patients on vitamin D supplementation (Table 1).

### 3.2. Biochemical Variables

**Figure 2:**
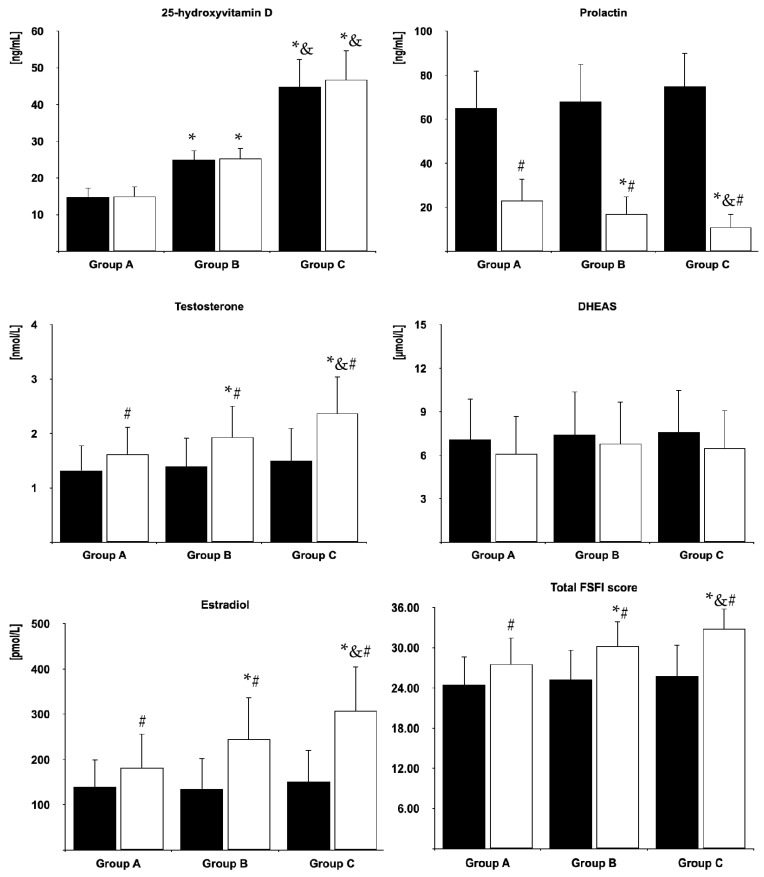
Biochemical variables and global sexual functioning in the study population. Group A: vitamin D-deficient women; Group B: vitamin D-insufficient women; Group C (control group): vitamin D-sufficient women. Black bars: before treatment; white bars: at the end of the study. * *p* < 0.05 vs. group A, ^&^ *p* < 0.05 vs. group B, ^#^ *p* < 0.05 vs. before treatment in the same study group.

Before cabergoline treatment, 25OHD levels were highest in group C and lowest in group A. There were no differences between baseline levels of prolactin, testosterone, DHEAS and estradiol between women with different vitamin D statuses. In all groups, cabergoline treatment reduced prolactin levels, increased testosterone and estradiol, and did not affect 25OHD and DHEAS. At the end of the study, prolactin levels were highest in group A and lowest in group C, while the opposite relationship was observed for 25OHD, testosterone and estradiol (Figure 2).

### 3.3. Sexual Functioning

**Figure 3:**
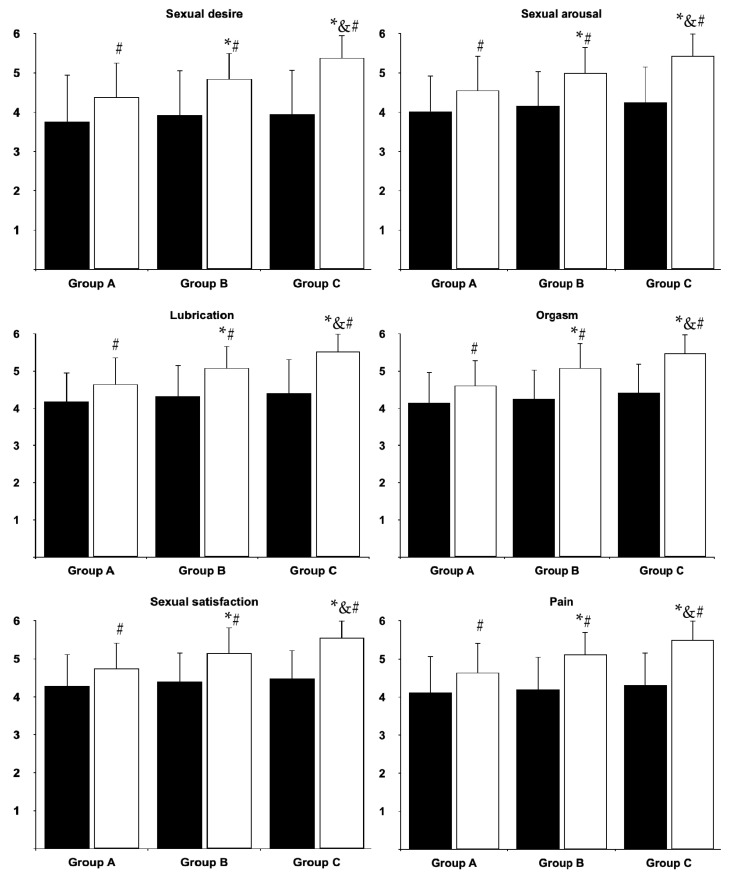
Different aspects of female sexual functioning in the study population. Group A: vitamin D-deficient women; Group B: vitamin D-insufficient women; Group C (control group): vitamin D-sufficient women. Black bars: before treatment; white bars: at the end of the study. * *p* < 0.05 vs. group A, ^&^ *p* < 0.05 vs. group B, ^#^ *p* < 0.05 vs. before treatment in the same study group.

At baseline, there were no differences in the total FSFI score and domain scores between the study groups. Sexual dysfunction was observed in 15 (63%) women in group A, 14 women in group B (58%) and 13 women (57%) in group C. Cabergoline treatment improved the overall FSFI score and domain scores for desire, arousal, lubrication, orgasm, sexual satisfaction and pain, as well as decreased the percentage of patients with sexual dysfunction in all study groups. After the six-month cabergoline treatment, the total score (Figure 2) and all domain scores (Figure 3) were lowest in group A and highest in group C. The opposite relationship was found for the percentage of patients with sexual dysfunction. At the end of the study, this complication was observed in 11 (46%) women in group A, seven women in group B (29%) and two women (9%) in group C.

### 3.4. Depressive Symptoms

At baseline, the overall BDI-II score and the percentage of patients with total and mild depressive symptoms did not differ between the study groups. Except for one patient in group A, there were no cases of moderate and severe depressive symptoms. Cabergoline decreased the BDI-II score and the percentage of patients with total and mild depressive symptoms only in group C. At the end of the study, the BDI-II score and the percentage of women with total and mild depressive symptoms were lower in group C than in the remaining groups (Table 2).

### 3.5. Correlations

**Figure 4:**
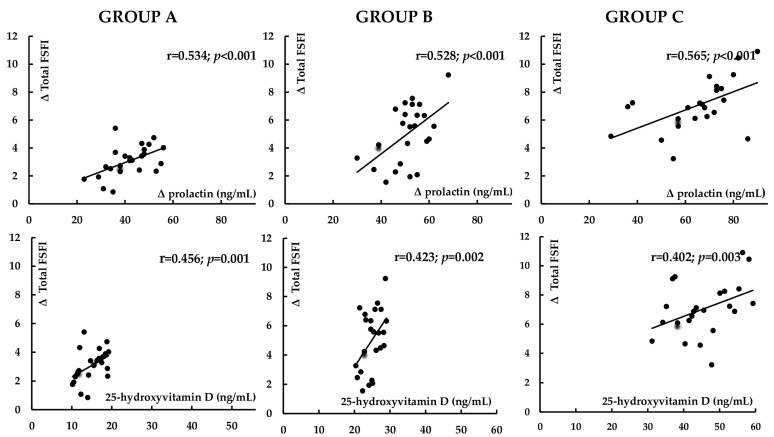
Correlations between the impact of cabergoline on female sexual functioning and on prolactin levels and between the improvement in female sexual functioning and vitamin D status. Group A: vitamin D-deficient women; Group B: vitamin D-insufficient women; Group C (control group): vitamin D-sufficient women. The data represent the correlation coefficient (r values).

The impact of cabergoline on prolactin levels positively correlated with baseline prolactin levels, baseline 25OHD levels, and the increase in testosterone and estradiol levels. There were also positive correlations between the cabergoline-induced increase in testosterone levels and baseline 25OHD. The effect of cabergoline on the total FSFI score and on all domain scores positively correlated with the reduction in prolactin levels and, except desire and arousal, with 25OHD concentration. There were positive correlations between the improvement in sexual desire and arousal and the increase in testosterone levels, as well as between the improvement in lubrication and pain and the increase in estradiol. The decrease in the overall BDI-II score positively correlated with the changes in the overall FSFI score and all domain scores, as well as with 25OHD levels (Figure 4, Table 3).

The impact of cabergoline on hormone levels, sexual functioning and depressive symptoms did not correlate with daily vitamin D intake and the percentage of patients on vitamin D supplements. The effects of cabergoline did not correlate with the reason for prolactin excess.

### 3.6. Multivariate Regression Analysis

Multivariate regression analysis evidenced that two factors were independently related to the improvement in sexual function: 25OHD levels (partial R^2^ between 0.219 (*p* = 0.008) and 0.262 (*p* < 0.001)) and the impact of cabergoline on prolactin levels (partial R^2^ between 0.231 (*p* = 0.006) and 0.312 (*p* < 0.001)). No biochemical variable was independently related to the severity of depressive symptoms.

## 4. Discussion

Our study provides some interesting observations. Unexpectedly, we did not observe differences in sexual function between women matched for prolactin levels with different vitamin D status. Thus, prolactin excess and disturbances in vitamin D homeostasis do not seem to have an additive effect on female sexual response. There are some potential explanations for this finding. Firstly, the unfavorable impact of hyperprolactinemia on female sexual function is much stronger than that of low vitamin D status, and may mask the impact of the latter. In line with this explanation, hyperprolactinemia disturbed all aspects of sexual response [4,5], while arousal, lubrication and pain were unaffected, even by severe vitamin D deficit [11]. Secondly, unfavorable changes in sexual functioning in response to prolactin excess and low vitamin D status may have common molecular underpinnings. In such a case, prolactin excess may mask the development of similar changes in the effector pathways in individuals with disturbed vitamin D homeostasis. This explanation may be supported by the lack of correlations between 25OHD and the total score and scores for the subscales of women’s sexual functioning before treatment. Thirdly, there may have been some differences in baseline characteristics, which counterbalanced the impact of low vitamin D status on female sexual health. However, matching the study groups argues against the role of age, BMI and blood pressure, playing a role in determining women’s sexual health [33,34,35]. Moreover, except for 25OHD, we have not identified other variables with different distributions in the study groups. Lastly, we cannot totally exclude that similar sexual functioning might have resulted from the moderate sample size and represented a type II error.

The multidirectional favorable impact of cabergoline treatment on all aspects of female sexual response in women with normal vitamin D homeostasis is in line with previous observations of our research team [9]. Owing to high specificity and affinity for the dopamine D_2_ receptor and a long elimination half-life (63–115 h), cabergoline is a potent inhibitor of prolactin [36]. Thus, the improvement in sexual functioning may be explained mainly by the reduction in prolactin levels. In line with this explanation, cabergoline markedly decreased its levels, and the increase in the total FSFI score and in all domain scores positively correlated with the impact on prolactin concentration. What is more, the changes in prolactin concentration were independently associated with the improvement in sexual functioning. Hence, post-treatment prolactin levels may serve as a predictor of the improvement in sexual functioning in this group of patients.

However, the most important observation of our research group was the inhibitory effect of low vitamin D status on female sexual function, likely reflecting a long-term deficit of vitamin D or its active metabolite. This finding does not seem to be explained by the pharmacological effects of exogenous vitamin D_3_, which is supported by several observations. Firstly, treatment-induced changes in the overall FSFI score and most domain scores inversely correlated with 25OHD levels. In the same study group, the strength of cabergoline action did not differ between women taking and not taking vitamin D_3_ supplements. Although the study groups differed in total daily vitamin D intake and in the percentage of users of vitamin D_3_ supplements, the impact of cabergoline on sexual function did not depend on either of these parameters. Lastly, a long interval between taking vitamin D_3_ supplements and administration of cabergoline argues against pharmacokinetic interactions at the level of absorption or metabolism between the studied dopamine agonist and exogenous vitamin D or other tablet ingredients.

The obtained results allow us to draw some practical conclusions. Firstly, positive correlations between cabergoline action on sexual function and on prolactin levels and between the decrease in this hormone and baseline prolactin levels suggest that the improvement in female sexual response may be greatest in women with severe hyperprolactinemia. Secondly, the impact of cabergoline on sexual functioning is unrelated to the reason for prolactin excess (it was observed in patients with microprolactinoma, iatrogenic prolactin excess, posttraumatic brain injury, empty sella syndrome and idiopathic hyperprolactinemia). Thirdly, the unfavorable influence of low vitamin D status on behavioral effects of cabergoline in reproductive-age women is not irreversible, and subsides in effectively supplemented patients. Fourthly, vitamin D status should be assessed in all cabergoline-treated women with sexual dysfunction persisting despite a remarkable reduction in prolactin levels. Fifthly, assessment of 25OHD before cabergoline treatment should be considered if impaired sexual functioning is the main indication for administration of a dopamine agonist. Lastly, we did not find any case of hypersexuality, compulsive shopping, binge eating or gambling, reported previously in males receiving dopamine agonists [37]. Thus, the risk of impulse control disorders in cabergoline-treated hyperprolactinemic women seems to be negligible.

An unexpected finding of our study was the lack of correlations between the impact of cabergoline on sexual desire and arousal and 25OHD levels. The improvement in these domains, however, positively correlated with testosterone levels, which increased in response to cabergoline treatment in women with normal vitamin D homeostasis and, though to a lesser degree, in women with vitamin D insufficiency and vitamin D deficiency. Moreover, the impact on testosterone positively correlated with 25OHD. Thus, the unfavorable interaction between low vitamin D status and cabergoline action on libido and arousal in women with hyperprolactinemia is likely to be, at least partially, mediated by testosterone. Interestingly, testosterone plays a more important role in regulation of drive and excitement than in the control of other aspects of female sexual response [38,39], which is in line with our explanation. Unlike males [40], no previous study investigated androgen production in women with non-elevated testosterone levels exposed to vitamin D or its active metabolite. However, in healthy non-obese women, 25OHD concentration was found to positively correlate with both testosterone and the free androgen index (an estimate of the biologically active testosterone [38]). Thus, both our study and the study by Chang et al. [41] may indicate that vitamin D potentiates testosterone production in women, and that this effect is mediated by the vitamin D receptor in the theca cells, which are responsible for ovarian androgen production [42]. In case of low vitamin D levels, this additive effect may be weak or even absent.

Another finding worth noting is the presence of correlations between the improvement in lubrication and pain and the increase in estradiol levels. Interestingly, both domains were found to be specific aspects of sexuality negatively affected by menopause, and their age-related worsening was probably secondary to a dramatic decrease in estradiol production [43]. The increase in estradiol levels in our study may be explained by activation of the hypothalamic–pituitary–ovarian axis in response to the reduction in plasma prolactin, which was more pronounced in women with normal than in women with low vitamin D status. Interestingly, we did not observe correlations between cabergoline action on the total and domain scores of the FSFI questionnaires and concentrations of DHEAS, which were stable throughout the study. DHEAS, a marker of the zona reticularis function, is characterized by the lack of significant diurnal, menstrual and seasonal cycles [39]. The neutral effect on this parameter and the lack of associations with domain scores suggest that the zona reticularis does not seem to play a role in mediating cabergoline action on female sexual function, irrespective of vitamin D status. Considering the young age of the study population and a short duration of hyperprolactinemia, the observed disturbances in female sexual response do not seem to be related to the changes in blood flow through genital organs and to peripheral and/or autonomic neuropathy.

As far as we know, very few studies have assessed the impact of dopamine agonists on depressive symptoms. Mild depression associated with prolactin excess in women was ameliorated by bromocriptine (but not by placebo) [44]. The same drug administered to women with mild hyperprolactinemia unselected for 25OHD levels insignificantly reduced the overall BDI-II score and the percentage of women with total and mild depressive symptoms [8]. In turn, the anti-depressant properties of cabergoline have been reported so far only in animals [45]. Thus, the current study is the first to show that cabergoline may improve mood in women with prolactin excess, and that the effect of any dopamine agonist on depressive symptoms is absent in women with even mild vitamin D deficit. Considering that vitamin D deficiency and insufficiency are frequently reported in reproductive-age women living in Poland [46], low vitamin D status may partially explain why the effect of bromocriptine in our previous study did not reach the level of statistical significance [8]. Because the impact on the BDI-II score, even in vitamin D-sufficient women, was relatively small, hyperprolactinemic women with moderate or severe depressive symptoms should probably additionally receive antidepressive drugs.

Positive correlations between the impact of cabergoline on BDI-II and on FSFI scores indicate that both these effects are reciprocally interrelated. Although the direction of this relationship remains unclear, our findings may suggest that disturbances in sexual functioning may contribute to mood worsening. In line with this explanation, unlike FSFI scores, we did not observe correlations between the impact on the overall BDI-II score and the changes in circulating levels of testosterone, DHEAS, estradiol, and, above all, prolactin. Moreover, no biochemical variable was an independent determinant of the BDI-II score. The relative strength of correlations indicates that lubrication and pain probably play a greater role in cabergoline-induced improvement in mood than the remaining aspects of female sexual response. The observed correlations between the impact on depressive symptoms and 25OHD levels suggest that cabergoline and vitamin D may additionally interact at the level of brain areas playing a role in the pathogenesis of depression. Interestingly, these brain structures—the hypothalamus, thalamus, prefrontal cortex and hippocampus—express the vitamin D receptor, 1α-hydroxylase (an enzyme required for the production of the active form of vitamin D) and D_2_ dopamine receptors [47,48]. Hence, low local availability of calcitriol may modulate cabergoline action in these brain structures, attenuating its impact on mood. This may explain why correlations between female sexual response and depressive symptoms were moderate.

Despite obtaining interesting findings, several study limitations should be pointed out. Because of the sample size, smaller differences might have gone undetected. Due to the cohort nature, the study was susceptible to selection and attrition biases. Owing to the inclusion criteria, it remains to be elucidated whether vitamin D status determines cabergoline action on sexual function and mood in postmenopausal women. Self-reported measures might have been influenced by human error, subjectivity or intentional misrepresentation. Although women with overt comorbidities were not considered for enrollment, preclinical disorders might have been present in some patients, potentially affecting sexual function and mood. Lastly, the study does not provide molecular mechanisms lying at the foundation of our findings.

## 5. Conclusions

**Figure 5:**
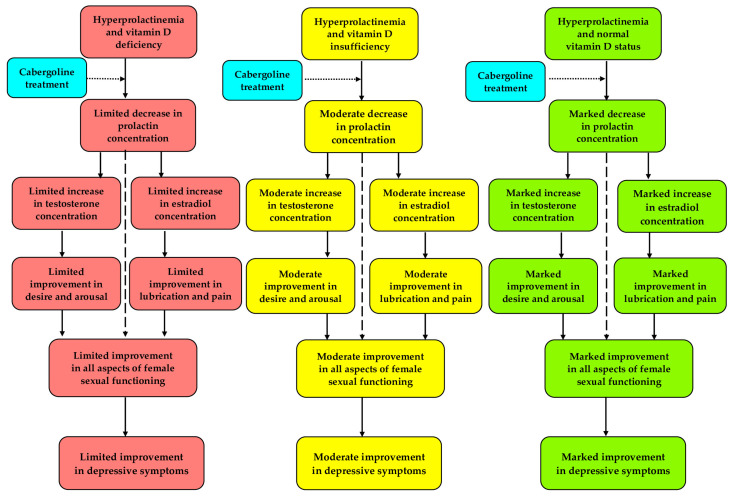
A hypothetical mechanism explaining the link between vitamin D status and cabergoline action on sexual functioning and depressive symptoms in young women with hyperprolactinemia.

Vitamin D status does not determine female sexual functioning and depressive symptoms in untreated reproductive-age women with prolactin excess. However, the improvement in sexual function in response to cabergoline treatment is less expressed in patients with vitamin D deficit, and inversely correlates with its severity. Clinically unfavorable interactions between low vitamin D status and cabergoline action on sexual functioning are partially mediated by testosterone and estradiol. Even mild disturbances in vitamin D homeostasis may attenuate the beneficial effect of cabergoline on depressive symptoms. The obtained results seem to indicate that vitamin D status determines the impact of dopamine agonists on female sexual response and mood (Figure 5). Thus, hyperprolactinemic women with sexual dysfunction may require normalization of vitamin D homeostasis to optimize the benefits resulting from cabergoline treatment. Because of their novelty and limitations of the study design, our findings require validation in prospective, randomized, placebo-controlled studies with a larger sample size. Future interventional studies should also assess whether simultaneous supplementation of vitamin D improves response to cabergoline treatment and whether the impact on sexual functioning and depressive symptoms differs between vitamin D_2_ and D_3_. Lastly, it would be important to better understand the molecular mechanisms underlying the interaction between prolactin and vitamin D.

## Figures and Tables

**Table 1:** General characteristics of the study population.

Variable	Group A	Group B	Group C
Number of patients	24	24	23
Age (years)	32 ± 6	31 ± 7	32 ± 6
Body mass index (kg/m^2^)	23.5 ± 4.9	23.2 ± 5.1	22.8 ± 4.7
Smokers (%)/Number of cigarettes a day (*n*)/Duration of smoking (months)	38/9 ± 7/95 ± 40	42/9 ± 6/89 ± 38	43/10 ± 7/85 ± 35
Reasons for prolactin excess: drug-induced hyperprolactinemia/microprolactinoma/brain injury/empty sella syndrome/idiopathic hyperprolactinemia	38/25/17/17/4	38/25/17/12/8	35/26/17/13/9
Physical activity: total/several times a week/once a week/once a month (%)	100/46/46/8	96/42/46/8	95/43/43/9
Primary or vocational/secondary/university education (%)	12/38/50	12/42/46	9/39/52
Occupational activity/Blue-collar/white-collar/pink-collar workers (%)	88/21/42/25	88/17/42/29	87/22/43/22
Number of sexual partners (*n*)	2.7 ± 1.0	2.5 ± 1.0	2.4 ±0.9
Number of marriages (*n*)/duration of marriages (months)	1.1 ±0.5/42 ± 15	1.2± 0.5/40 ± 15	1.1 ± 0.6/38 ± 12
Number of deliveries (*n*)/Number of miscarriages (*n*)	1.3 ± 0.6/0.5 ± 0.5	1.2 ± 0.6/0.5 ± 0.5	1.2 ± 0.5/0.7 ± 0.5
Stress exposure (%)	79	83	78
Systolic blood pressure (mm Hg)	125 ± 16	123 ± 17	119 ± 15
Diastolic blood pressure (mm Hg)	78 ± 6	77 ± 5	76 ± 6
Total daily vitamin D intake (µg)	8.0 ± 2.8	12.7 ± 3.8 ^&^	26.8 ± 8.6
Users of vitamin D_3_ supplements (%) ^1^	4 *^&^	38 ^&^	57

Group A: vitamin D-deficient women; Group B: vitamin D-insufficient women; Group C (control group): vitamin D-sufficient women. Unless otherwise stated, the data have been shown as the mean ± standard deviation. ^1^ no patient received vitamin D_2_ supplements. * *p* < 0.05 vs. group B, ^&^ *p* < 0.05 vs. group C.

**Table 2:** Depressive symptoms in the study population.

Variable	Group A	Group B	Group C
BDI-II score (mean ± standard deviation)			
*Before treatment*	13.7 ± 3.2	13.0 ± 2.8	12.6 ± 2.9
*At the end of the study*	12.9 ± 3.1	12.1 ± 3.2	10.2 ± 3.0 *^&#^
Depression symptoms (*n* [%])			
*Before treatment*	13 [54]	12 [50]	10 [43]
*At the end of the study*	11 [46]	10 [42]	5 [21] *^&#^
Mild symptoms (*n* [%])			
*Before treatment*	12 [50]	12 [50]	10 [43]
*At the end of the study*	11 [46]	10 [42]	5 [21] *^&#^
Moderate symptoms (*n* [%])			
*Before treatment*	1 [4]	0 [0]	0 [0]
*At the end of the study*	0 [0]	0 [0]	0 [0]
Severe symptoms (*n* [%])			
*Before treatment*	0 [0]	0 [0]	0 [0]
*At the end of the study*	0 [0]	0 [0]	0 [0]

* *p* < 0.05 vs. group A, ^&^ *p* < 0.05 vs. group B, ^#^ *p* < 0.05 vs. before treatment in the same study group.

**Table 3:** Correlations between the outcome measures.

Correlated Variables		Group A	Group B	Group C
Δ Prolactin	Prolactin	0.563 ***	0.588 ***	0.655 ***
Δ Prolactin	25OHD	0.487 ***	0.447 **	0.418 **
Δ Prolactin	Δ Testosterone	0.315 *	0.325 *	0.341 *
Δ Prolactin	Δ Estradiol	0.542 ***	0.567 ***	0.602 ***
Δ Testosterone	25OHD	0.422 **	0.448 **	0.456 **
Δ Sexual desire	Δ Prolactin	0.412 **	0.395 **	0.408 **
Δ Sexual arousal	Δ Prolactin	0.388 **	0.411 **	0.425 **
Δ Lubrication	Δ Prolactin	0.312 *	0.385 **	0.342 *
Δ Orgasm	Δ Prolactin	0.515 ***	0.532 ***	0.608 ***
Δ Sexual satisfaction	Δ Prolactin	0.568 ***	0.594 ***	0.572 ***
Δ Pain	Δ Prolactin	0.353 *	0.324 *	0.305 *
Δ Sexual desire	25OHD	0.168	0.155	0.175
Δ Sexual arousal	25OHD	0.184	0.122	0.103
Δ Lubrication	25OHD	0.455 **	0.421 **	0.397 **
Δ Orgasm	25OHD	0.462 **	0.442 **	0.411 **
Δ Sexual satisfaction	25OHD	0.432 **	0.421 **	0.388 **
Δ Pain	25OHD	0.412 **	0.376 **	0.354 *
Δ Sexual desire	Δ Testosterone	0.483 ***	0.523 ***	0.551 ***
Δ Sexual arousal	Δ Testosterone	0.462 **	0.487 ***	0.514 ***
Δ Lubrication	Δ Estradiol	0.415 **	0.420 **	0.437 **
Δ Pain	Δ Estradiol	0.429 **	0.452 **	0.429 **
Δ BDI-II	Δ Total FSFI score	0.345 *	0.364 *	0.359 *
Δ BDI-II	Δ Sexual desire	0.384 *	0.392 **	0.408 **
Δ BDI-II	Δ Sexual arousal	0.402 **	0.428 **	0.342 **
Δ BDI-II	Δ Lubrication	0.646 ***	0.594 ***	0.602 ***
Δ BDI-II	Δ Orgasm	0.423 **	0.405 **	0.317 *
Δ BDI-II	Δ Sexual satisfaction	0.385 **	0.427 *	0.406 *
Δ BDI-II	Δ Pain	0.622 ***	0.586 ***	0.614 ***
Δ BDI-II	25OHD	0.478 ***	0.457 **	0.443 **

Group A: vitamin D-deficient women; Group B: vitamin D-insufficient women; Group C (control group): vitamin D-sufficient women. The data represent the correlation coefficients (r values). * *p* < 0.05, ** *p* < 0.01, *** *p* < 0.001.

## Data Availability

The data that support the findings of this study are available from the corresponding author upon reasonable request.
